# Use of automated conversational agents in improving young population mental health: a scoping review

**DOI:** 10.1038/s41746-024-01072-1

**Published:** 2024-03-19

**Authors:** Raluca Balan, Anca Dobrean, Costina R. Poetar

**Affiliations:** 1 The International Institute for the Advanced Studies of Psychotherapy and Applied Mental Health, Babeș-Bolyai University, Cluj-Napoca, Romania; 2 Department of Clinical Psychology and Psychotherapy, Babeş-Bolyai University, Cluj-Napoca, Cluj Romania

**Keywords:** Human behaviour, Diseases

## Abstract

Automated conversational agents (CAs) emerged as a promising solution in mental health interventions among young people. Therefore, the objective of this scoping review is to examine the current state of research into fully automated CAs mediated interventions for the emotional component of mental health among young people. Selected databases were searched in March 2023. Included studies were primary research, reporting on development, feasibility/usability, or evaluation of fully automated CAs as a tool to improve the emotional component of mental health among young population. Twenty-five studies were included (*N* = 1707). Most automated CAs applications were standalone preventions targeting anxiety and depression. Automated CAs were predominantly AI-based chatbots, using text as the main communication channel. Overall, the results of the current scoping review showed that automated CAs mediated interventions for emotional problems are acceptable, engaging and with high usability. However, the results for clinical efficacy are far less conclusive, since almost half of evaluation studies reported no significant effect on emotional mental health outcomes. Based on these findings, it can be concluded that there is a pressing need to improve the existing automated CAs applications to increase their efficacy as well as conducting more rigorous methodological research in this area.

## Introduction

Mental health problems are an area of particular concern among young people. According to WHO, 20% of youths have a mental health disorder, a rate that is two times higher than in the general population^1^. A history of mental health problems in young age forecasts a range of psychosocial difficulties in adult life^2^. Despite high prevalence and long-term negative consequences of mental health problems, most children and youths do not participate in preventive or intervention actions because of attitudinal or logistic barriers^3^.

Use of technology has emerged as an important alternative to face-to-face approach in deploying assistive, preventive, and therapeutic solutions for those in need, increasing the availability and providing a stigma free environment for exploring their vulnerabilities related to mental health problems^4^. One such cutting edge digital solution is conversational agents (CAs), defined as systems simulating human interaction using text, speech, gestures, facial, or sensorial expressions as input and/or output^5^. The category of CAs covers a broad spectrum of embodiment types, from disembodied agents with no dynamic physical representation (chatbots) to agents with virtual representation or robots with a physical representation^6^. The autonomy level ranges from non-autonomous CAs, whose functionality totally depends on the decisions and actions of a human being, to semi-autonomous CAs (that have a certain degree of independence but require the real time control by humans for some specific scenarios and functionalities) to fully automated CAs, that can be used totally independently without any form of human support^7^. In this paper, the focus will be on fully automated CAs, irrespective of embodiment type.

With a rapid technological expansion, fully automated CAs seem to hold a great potential in mental health care for young people. In recent years, a growing body of research has been interested in developing and testing the efficacy of fully automated CAs for addressing mental health problems in a variety of settings with youths. In the healthcare setting, automated CAs are used to tackle distress related to medical procedures among youths, such as vaccination or cancer treatments^8,9^. In an educational context, they have been employed as a tool to reduce problems such as general distress or performance anxiety^10,11^. Automated CAs have also been used to prevent or to treat depression and anxiety in the general or psychiatric population^12^.

While several reviews have been conducted to characterize various types of CAs as tools for treatment of mental health problems, several limitations have been identified. First, the previous reviews rely mostly on the adult population or do not distinguish between young and older population, with no comprehensive synthesis of existing automated CAs specifically designed to tackle mental health problems among young populations^13–16^. Justification for focusing on the young population is rooted in prior research demonstrating distinctive preferences, attitudes, and utilization patterns compared to adults^17,18^. As first adopters of the latest technological developments, including mental healthcare services, youths exhibit greater familiarity and comfort with these innovations^19^.

Second, most of the previous reviews did not distinguish fully automated CAs from non- or semi-autonomous CAs^13,20^. Fully automated CA are a scalable, cost effective and alternative to human therapist support, moving the field towards a new paradigm. However, full automatization can pose significant challenges when used in mental health care with youths, such as limited capacity to respond to safety-critical situations, less personalization of the content or confidentiality issues^21,22^.

Third, the previous reviews limited their focus to a subset of CAs based on the embodiment level, such as disembodied CAs^13,20^, CAs with virtual representation^15^, or with a physical representation^23–25^. Moreover, use of CAs was predominantly investigated in relation to a broad range of mental health problems^15,16^, or specifically related to cognitive and social abilities, without considering the emotional component of mental health^24,25^. This scoping review was formulated to focus specifically on the emotional component of mental health as defined through the lens of the medical model (e.g., changes in anxiety, depression, psychological distress) rather than social (e.g., repertoire of verbal/non-verbal abilities to communicate and interact with others) and cognitive skills (e.g., executive functioning skills) to specifically capture this innovative and growing application area for automated CAs.

In response to these gaps, this scoping review aims to provide a comprehensive overview of fully automated CAs and their role in enhancing the emotional component of mental health in the young population. The scoping review was guided by the following research questions:

1. What are the technological characteristics of automated CAs used to deliver interventions for youth’s mental health?
2. What are the characteristics of the interventions provided by automated CAs in children, adolescents, and young adults aiming to improve mental health outcomes?

## Results

### Study selection

**Fig. 1:**
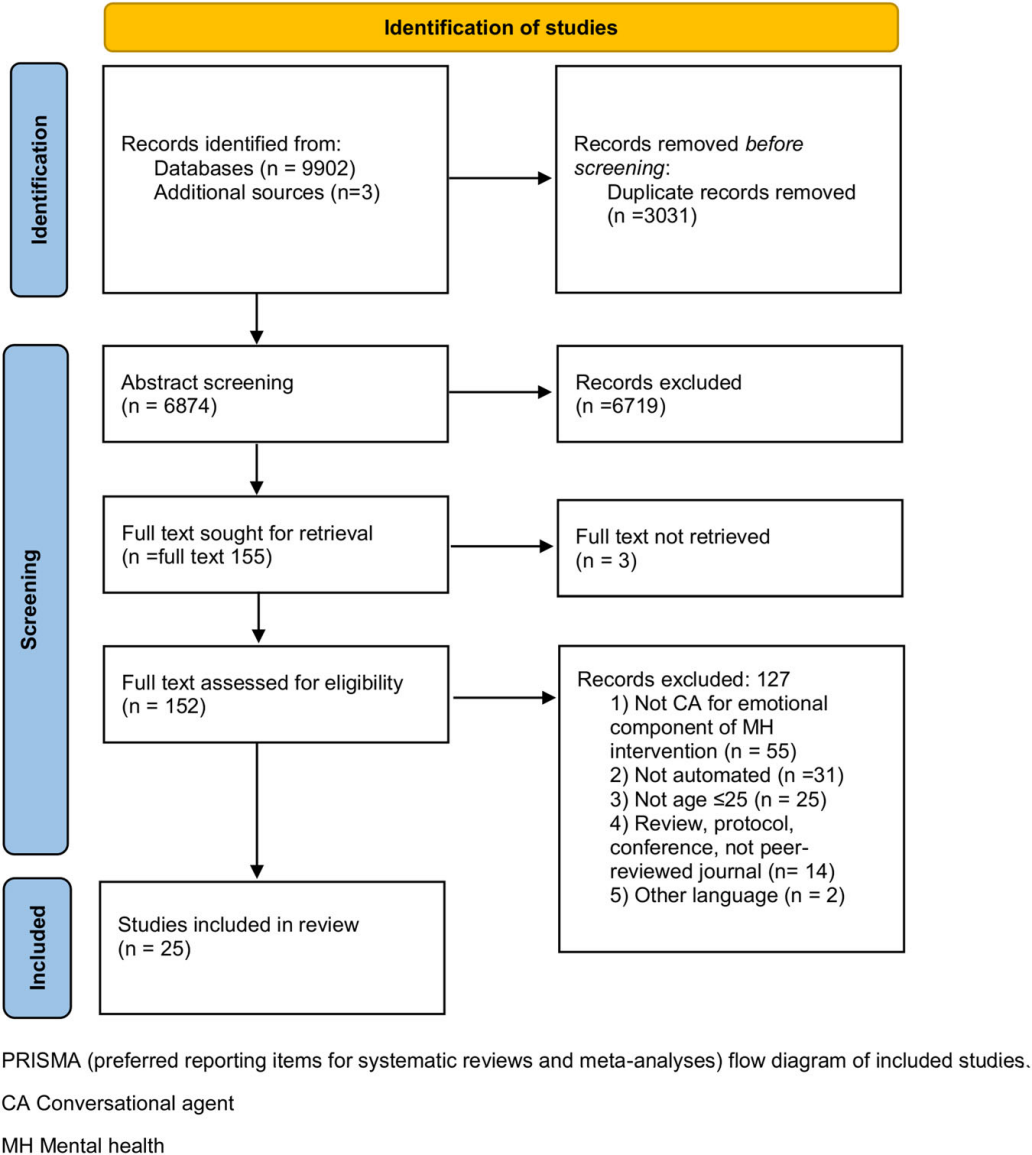
PRISMA flow.

The systematic search in databases and external sources returned 9905 articles. After duplicates removal, 6874 articles were screened for title and abstract and further 6719 studies were excluded. Out of the remaining 155 studies, we retrieved full-text copies for 152 articles that were screened in full. This resulted in a total of 25 studies included in the current scoping review. The study selection is detailed in Fig. 1 PRISMA flowchart.

A detailed overview of characteristics of included studies is provided in Supplementary Table 1 and 2.

Of the 25 studies, 19 were recently published (between 2020 and 2023)^8–10,12,26–40^. Studies were conducted predominantly in the US (*n* = 12)^11,12,27,28,33,34,38,41–45^, followed by Europe (*n* = 5)^10,26,29,32,35^, Asia (*n* = 4)^9,31,36,39^, New Zealand (*n* = 2)^37,40^, and Australia (*n* = 1)^30^.

### Technological characteristics

The summative results for technological characteristics of automated CAs are presented in Table 1. In total, there were 21 different agents described in the included studies. Only 3 of the CA were the focus of more than one study – Paro^11,41,45^, Nao^8,38^, and Woebot^12,42^. These automated CAs were predominantly disembodied chatbots (*n* = 15)^10,26,28–32,35–37,40,42–44^, followed by robots (*n* = 7)^8,9,33,34,38,41,45^. Automated CAs with a virtual representation were the focus in 2 studies^11,27^. In addition, one application consisted of a chatbot with features of avatar^39^.

**Table 1:** Summative results per technological characteristics

Category	Number of studies	Percentage	Studies
Type of embodiment			
Disembodied	15	60%	^10,26,28–32,35–37,40,42–44^
Virtual representation	2	8%	^11,27^
Physical representation	7	28%	^8,9,33,34,38,41,45^
Combined	1	4%	^39^
Dialog system			
Rule-based	10	40%	^10,11,26,28,29,32,33,35,40,44^
AI-based	12	48%	^8,9,12,27,30,31,34,38,39,41,43,45^
Mixed	3	12%	^36,37,42^
Modality of communication			
Text	13	54.1%	^10,12,26,28–30,32,35,37,40,42–44^
Speech	2	8.3%	^8,38^
Non-verbal	4	16.7%	^9,34,41,45^
Multimodal	5	20.8%	^11,31,33,36,39^
Availability			
Yes	17	70.8%	^8–10,12,27,31,33,34,36–38,40–45^
No	7	29.2%	^11,26,28–30,32,35^

Regarding the dialog system underlying the process of conversation, almost half of automated CAs (*n* = 12) employed natural language processing and machine learning to carry on an interaction^8,9,12,27,30,31,34,38,39,41,43,45^. Predefined dialog or interactions assembled, and matched to the user input in a dynamic manner was used in 10 studies^10,11,26,28,29,32,33,35,40,44^, while 3 used a mixed dialog system^36,37,42^. These agents communicated through text (*n* = 13)^10,12,26,28–30,32,35,37,40,42–44^, speech (*n* = 2)^8,38^, and non-verbal cues (*n* = 4)^9,34,41,45^, while multiple modalities communication was employed by 5 studies^11,31,33,36,39^. For one study no information was provided on modality of communication^27^. Among automated CAs investigated in the included studies, 17 are available to purchase or for free use^8–10,12,27,31,33,34,36–38,40–45^.

### Characteristics of interventions

Characteristics of mental health interventions using automated CAs are detailed in Table 2.

**Table 2:** Summative results per characteristics of interventions

Category	Number of studies	Percentage	Studies
MH target			
Anxiety	12	48%	^8,10,12,27,33,35,37,38,41–43,45^
Depression	8	32%	^12,28,31,35,36,38,42,43^
General distress	5	20%	^9,10,34,39,40^
Mood	2	8%	^33,41^
Mental health problems	1	4%	^32^
Psychological well being	5	20%	^26,29,30,33,44^
Scope			
Prevention	17	68%	^21,23–26,28–31,33,35–37,42–45^
Intervention	8	32%	^8–11,26,28–30,32–35,37,38,43–45^
Duration			
1 day or less	5	26.3%	^9,28,34,41,45^
2−7 days	3	15.8%	^21,26,33^
2−4 weeks	8	42.1%	^8,10,29,38–40,42–44^
More than 4 weeks	3	15.8%	^12,35,36^
Frequency	8		
Daily	3	37.5%	^26,33,43^
3 times per week	1	12.5%	^35^
Bi-weekly	3	37.5%	^10,29,43^
Once per week	1	12.5%	^39^
Independence			
Standalone	20	80%	^8–10,26,28–34,36–38,40–45^
Component	5	20%	^11,12,27,35,39^
Theoretical framework			
CBT	14	82.4%	^8,10–12,26,28,31,35–37,42–44^
PP	5	20%	^29,33,37,40,44^
ITP	1	4%	^12^
PCT	1	4%	^39^
MINI	1	4%	^38^
MI	1	4%	^43^
TM	1	4%	^43^
EFT	1	4%	^43^
DBT	1	4%	^12^

*CBT* Cognitive Behavioral Therapy, *DBT* Dialectical and Behavioral Therapy, *EFT* Emotion focused therapy, *IPT* Interpersonal Therapy, *MI* Motivational Interview, *MINI* Metacognitive Intervention of Narrative Imagery, *MH* Mental Health, *PCT* Person Centered Therapy, *PP* Positive Psychology, *TM* Transtheoretical Model.

Anxiety was the most frequent targeted emotional component of mental health by automated CAs (*n* = 12)^8,10,12,27,33,35,37,38,41–43,45^. Depression was the second most targeted emotional component (*n* = 8)^12,28,31,35,36,38,42,43^, followed by psychological well-being (*n* = 5)^26,29,30,33,44^, general distress (*n* = 5)^9,10,34,39,40^, and mood (*n* = 2)^33,41^. One intervention had as target mental health problems as a broad construct^32^.

With respect to the scope of interventions, most of the studies labeled the CAs applications as interventions. In fact, those were designed and tested as having mainly a preventive scope, since the research was conducted with general or at-risk population^8–11,26,28–30,32–35,37,38,43–45^. Only 8 studies were conducted on samples of youths screened as having detectable mental health problems, mainly based on youth or parent report^12,27,31,36,40–42^.

Duration of interventions was reported by 19 studies. Most of the interventions last between 2- and 4-weeks (*n* = 8)^8,10,29,38–40,42–44^, followed by interventions with a duration of 1 day or less (*n* = 5)^9,28,34,41,45^, and interventions of 2 up to 7 days (*n* = 3)^26,31,33^. Only 3 studies investigated interventions longer than 4 weeks^12,35,36^. In terms of sessions’ frequency only 8 studies provide information and include daily sessions^26,33,43^, bi-weekly^10,29,43^, once a week^39^ or 3 times per week^35^.

Out of 25 included studies, only 5 focused on automated CAs as components embedded in other types of technologies or mental health services for mental health problems^11,12,27,35,39^. The remaining 20 studies designed or evaluated automated CAs agents as standalone psychological interventions. Automated CAs that were not independent interventions were integrated components of web-based interventions, with additional technological features enabling the intervention such as videoconference or serious games^11,27,35,39^ or as an additive component to primary care management^12^.

Theoretical framework for automated CAs interventions was reported by 17 studies. Cognitive behavioral theory (CBT) principles were applied to most of the interventions to derive their content. More specifically, CBT was mentioned as a theoretical framework for 14 automated CAs applications^8,10–12,26,28,31,35–37,42–44^. Among CBT based interventions, 2 applications mentioned relying exclusively on the third wave of CBT principles—acceptance and commitment therapy (ACT)^26,35^. The second most reported theoretical framework was positive psychology, with 5 of automated CAs applications mentioning it as guiding theory for the content of the intervention^29,33,37,40,44^. Other theoretical frameworks were Interpersonal Theory^12^, Person Centered Theory^39^, Metacognitive Intervention of Narrative Imagery^38^, Motivational Interview^43^, Transtheoretical Approach^43^, Emotion Focused Theory^43^, and Dialectical Behavioral Theory^12^. The number of theoretical approaches guiding one intervention ranged from 1 to 4 (median 2.5).

### Characteristics of peer-reviewed research

Summative results for characteristics of peer reviewed research are presented in Table 3.

**Table 3:** Summative results per characteristics of peer reviewed research

Category	Number of studies	Percentage	Studies
Recruitment setting			
Community	6	27.3%	^26,28,34,37,41,44^
Educational	10	45.4%	^10,11,31,33,35,36,39,40,42,43^
Healthcare	6	27.3%	^8,9,12,27,38,45^
Health status			
Physical health condition	4	17.4%	^8,38,44,45^
Mental health condition	7	30.4%	^12,31,36,39–42^
Undergoing a medical procedure	2	8.7%	^9,27^
Any	10	43.5%	^10,11,26,28,29,33–35,37,43^
Sample size			
<50	9	39.1%	^12,26,28,29,33,38,39,44,45^
50−100	8	34.8%	^9,10,27,34,36,41–43^
>100	6	26.1%	^8,11,31,35,37,40^
Stage of research			
Development/design	2	8%	^30,32^
Feasibility/usability	1	4%	^28^
Evaluation	7	28%	^9,30,31,35,40,44,45^
Design and feasibility/usability	1	4%	^29^
Feasibility/usability and evaluation	12	48%	^10,12,26,27,31,33,36,38,40,42–44^
Design and evaluation	1	4%	^39^
Design, feasibility/usability, and evaluation	1	4%	^37^
Study design (evaluation and feasibility/usability)			
RCT	12	52.1%	^8,9,11,12,27,31,34–36,41–45^
Non-randomized controlled trial	2	8.7%	^9,27^
Uncontrolled trial pre post	7	30.4%	^10,26,33,37–40^
Uncontrolled trial post only	2	8.7%	^28,29^
Study design (development)			
Co-participatory design	4	100%	^30,32,37,39^
Study methodology			
Quantitative	8	32%	^8,9,11,34,35,38,41,45^
Qualitative	2	8%	^30,32^
Mixed methods	15	60%	^10,12,26–29,31,33,36,37,39,40,42–44^
Type of control group			
Passive control	3	21.4%	^11,12,34^
Active control	11	78.6%	^8,9,27,30,31,35,36,41–43,45^

Participants were predominantly recruited from an educational setting (*n* = 10)^10,11,31,33,35,36,39,40,42,43^, followed by community setting (*n* = 6)^26,28,34,37,41,44^, and hospital/healthcare settings (*n* = 6)^8,9,12,27,38,45^. Sample sizes ranged between 8 and 234 participants, with 9 studies conducted on samples of less than 50 participants^12,26,28,29,33,38,39,44,45^, 8 studies on samples between 50 and 100 participants^9,10,27,34,36,41–43^, and 6 studies on samples above 100 participants^8,11,31,35,37,40^. The presence of emotional problems on a certain level was required by 7 studies^12,31,36,39–42^, whereas 4 studies focused on physical health condition as selection criteria^8,38,44,45^. Additionally, undergoing a medical procedure, irrespective of health condition, was a selection criterion for 2 studies^9,27^. The mean age of participants was 16.64. Females represented 58.14% of the total sample size.

With respect to the stage of research, most studies fall under combinations of research stages: 12 studies on feasibility/usability and evaluation^10,12,26,27,31,33,36,38,40,42–44^, 1 on development and feasibility/usability^29^, 1 on design and evaluation^39^, and 1 on design, feasibility/usability, and evaluation^37^.

Among the 23 feasibility/usability and/or evaluation studies, more than half were controlled studies (*n* = 14)^8,9,11,12,27,31,34–36,41–45^. Controlled studies predominantly employed an active control group (*n* = 11)^8,9,27,30,31,35,36,41–43,45^. Among the studies reporting on design and development of automated CAs, 3 used co-participatory and iterative designs, involving the young end users in different stages of development^30,32,37^. One study reporting on development relied only on mental health specialists and researchers input in design^39^. The methodological approaches most frequently employed were mixed (*n* = 15)^10,12,26–29,31,33,36,37,39,40,42–44^ and quantitative methods (*n* = 8)^8,9,11,34,35,38,41,45^.

The feasibility/usability outcomes were reported in 15 studies and include parameters such as engagement, retention/adherence rate, acceptability, user satisfaction, usability of the system, safety, and functionality^10,12,26,27,31,33,36,38,40,42–44^. Overall, the feasibility and usability parameters were reported to be relatively high across studies. However, a few exceptions are worth mentioning. Safety issues were reported in 2 studies^12,26^. More than half of the participants reported at least one negative effect of the intervention delivered through SISU chatbot^26^. A serious adverse event occurred, 1 participant reporting suicidal tendency for the first time after intervention^26^. One study reported that during study participation, 4 (24%) participants had one alert for suicidal ideation 4 participants had 3, and 2 participants had 6. One parent from the intervention group reported in week 12 that his child was seen in an emergency department and discharged to go home^12^. With respect to engagement and adherence, 2 studies point out a decrease of these parameters over time^29,31^. The drop-out rates ranged between 0 and 70.9%.

All studies reporting evaluation outcomes included efficacy parameters (*n* = 21), with no study on cost-effectiveness. In terms of efficacy outcomes, almost half of the studies reported more than one mental health outcome. Summative results for efficacy outcomes per outcome and research design are presented in Table 4.

**Table 4:** Summative results for efficacy per outcome and study design

Outcome	Study design	Effect	Number of studies	Percentage	Studies
Anxiety	Controlled	Positive	6	60%	^12,33,36,43,45^ ^27a^
		No effect	4	40%	^11,35,41,42^
	Uncontrolled	Positive	3	60%	^36,38^ ^10^^b^
		No effect	1	20%	^40^
		Negative	1	20%	^26^
Depression	Controlled	Positive	5	71.4%	^12,31,36,42,43^
		No effect	2	28.6%	^35,44^
	Uncontrolled	Positive	1	50%	^38^
		No effect	1	50%	^26^
Negative affect	Controlled	Positive	1	16.7%	^43^
		No effect	5	83.3%	^34,36,41,42,44^
Positive affect	Controlled	Positive	3	50%	^34,41,43^
		No effect	3	50%	^36,42,44^
Distress	Controlled	Positive	2	100%	^8,9^
	Uncontrolled	Positive	2	66.7%	^38,39^
		Negative	1	33.3%	^10^^c^
Psychological wellbeing	Uncontrolled	Positive	2	100%	^33,40^
Subjective happiness	Uncontrolled	No effect	1	100%	^39^
Psychological sensitivity	Uncontrolled	Positive	1	100%	^39^
Physiological arousal	Uncontrolled	No effect	1	100%	^41^
Post-traumatic stress disorder	Uncontrolled	No effect	1	100%	^26^

^a^Only for only for participants undergoing more invasive procedures and with more frequent exposure to medical procedures.

^b^only for participants with initial high levels of anxiety.

^c^only for participants with initial high distress scores.

Anxiety outcomes were reported in 15 studies. When comparing the effect of automated CAs with a control group on anxiety measures, 5 studies reported a positive significant difference compared to control, favoring the automated CA condition^12,33,36,43,45^, whereas 4 studies found no significant difference^11,35,41,42^. One RCT found an improvement in medical procedure related anxiety only for a subgroup of participants, namely those undergoing more invasive procedures and with more frequent exposure to medical procedures^27^. Among uncontrolled studies, a significant decrease in anxiety from baseline to post-intervention was reported in 2 studies^36,38^, no effect in one study^40^, while one study reported a negative effect of the automated CA mediated intervention expressed as an increase in anxiety symptoms^26^. One uncontrolled study reported a significant decrease in anxiety only for youths with initial high levels of anxiety^10^.

Depression was reported in 9 studies. Among controlled trials focusing on reducing depression, 5 studies reported a significant difference between control and automated CA group, favoring the experimental condition^12,31,36,42,43^, whereas 2 controlled studies found no significant difference on depression scores^35,44^. Among uncontrolled trials, a minimal change in depression score was reported in one study using a robot^38^, whereas another study showed no improvement from pre to post test^26^.

Positive and negative affect were separately assessed in 6 studies^34,36,41–44^, whereas one study used a composite measure of overall affect, combining both facets in one score^33^. All but one study^43^ reported no significant difference between control group and automated CA condition in reducing negative affect. However, an improvement in positive affect was found in 3 studies^34,41,43^, while the other 3 remaining studies reported no difference between groups on this outcome^36,42,44^. In one study, a robot coach delivering a positive psychology intervention improved the overall affect among young adults^33^.

The effect of automated CAs mediated intervention on distress was explored in 5 studies. Out of the 5 studies, 2 used a controlled design and found a significant effect on distress after 5- and 20-min post-intervention, but not immediately following the intervention^8,9^. Among uncontrolled studies, 2 studies report a significant decrease in distress outcomes from pre to post intervention^38,39^, while other study found a significant effect on distress only for participants with initial high distress scores^10^ Moreover, a negative effect was reported for those with initial low levels of distress, for whom distress increased from pre to post intervention^10^.

Two uncontrolled studies were conducted to test the effectiveness of automated CAs mediated intervention on psychological well-being, showing a significant improvement^33,40^. One study reported as outcome a measure of psychological sensitivity, which also showed a significant decrease from pre- to post-intervention^39^. No significant effect of a chatbot based intervention on subjective happiness was reported in the uncontrolled study^39^. An indicator of anxiety—physiological arousal—was reported in one study, with no change from pre- to post-intervention^41^. Similarly, post-traumatic stress disorder symptoms showed no significant improvement after an agent-based software intervention^26^.

## Discussion

The field is marked by a notable surge in the deployment of fully automated CAs specifically designed to address the emotional facets of mental health in the youth, with our review scrutinizing 21 distinct automated CAs across 25 included papers. Considering that most of these studies were published between 2020 and 2023, it is evident that the literature in this realm is still in its early stages. Despite the potential to extend support to a larger demographic of the young population, our findings underscore a significant lag in the adoption of automated CA-mediated interventions in less developed countries. The deployment of such entities typically incurs substantial financial outlays, a factor that inherently influences their accessibility and widespread adoption. This economic consideration is a critical aspect in understanding the differential integration of these technologies, particularly in contexts where resource allocation plays a pivotal role. However, there was an expansion of digital application in mental health and of shipped phones – that can be used to access at least text based and speech automated CAs available in the commercial market, therefore more research in other geographic areas is expected to be conducted^46^.

The technological capabilities of automated CAs interventions for youths are evolving from simple oriented tasks and predefined decision trees to more complex and interactive solutions, as shown by the predominance of AI-based technologies. However, the state-of-the-art lags in terms of other technological capabilities such as embodiment and communication channels. This aspect holds particular significance, as previous research indicated that youths exhibit improved responsiveness and greater openness to CAs that possess virtual or physical representation, in contrast to disembodied CAs^47^. Furthermore, although young people are used to typing and text messaging, there is evidence pointing to youth preference towards an interaction with CAs using speech and auditory channels beyond text^48^. Similar conclusions were drawn by reviews conducted with the adult population in clinical psychology and healthcare with respect to the status of CAs technological capabilities, showing a rapid development in terms of dialog systems employed but a slower progress related to other technological capabilities such as type of representation and communication^14,20^. However, while adults’ acceptability of CAs might revolve around less sophisticated and thus more familiar technologies, youths hold higher expectations since they learn and adopt new cutting-edge technologies from their infancy. Therefore, these aspects might weigh more for youths than adults when it comes to the acceptability and uptake of current automated CAs as mental health solutions.

The prevailing focus of current automated CAs mediated interventions centers on mitigating emotional problems, leaving limited attention to fostering positive aspects of emotional mental health, such as happiness or psychological well-being. A recent study showed that youth’s preference regarding psychological interventions for emotional problems revolves around a balance between the medical model of mental health, oriented to solving problems and the growth positive models, based on the assumption that all human beings have the capacity to flourish, and build upon existing strengths^49^. This might be more relevant when it comes to appealing technologies such as automated CAs, since it is possible that youths make an indirect association between the appealing, interactive tool and positive aspects in its content.

Our review emphasizes an advanced stage of research development, with a predominance of a combination of feasibility/usability and evaluation studies, conducted as controlled trials using an active control condition. This contrasts with research conducted on subsets of CAs or with adults, that identified mainly pilot uncontrolled studies investigating their feasibility and usability^20^. However, as shown by the other reviews, the stage of system design and development of automated CAs mediated intervention as well as the input from end users from initial stages is often neglected^14,15^. Relatively little attention has been given to the investigation of a priori preference of end users in terms of scope, features, personality, and content and to the use of the results to inform the development of the automated CAs from early stages^26,28^. This is in contradiction with the advocated human centered approaches, that have the potential to enhance the uptake of CAs as mental health digital solutions^50,51^.

The existing automated CAs appear to hold possibilities to support youths’ mental health mainly in community settings and less in clinical context. While previous reviews on adults show a growing use of CAs in treatment of mental health problems, the evidence supporting applicability of automated CAs in improving emotional health among youths is limited to non-clinical populations^8^. However, the broad spectrum of the care sector, ranging from healthcare applications to providing emotional support during medical procedures, as well as educational contexts addressing anxiety and distress, reflects the versatile potential of automated CAs. Nevertheless, our review highlights a scarcity of applications targeted at younger children, potentially attributed to the fully autonomous nature of the CAs reviewed, requiring human facilitation. Furthermore, our investigation revealed a discernible pattern associating distinct types of embodiments with specific emotional challenges and age groups. Notably, automated CAs with physical embodiments demonstrated enhanced relevance in addressing transient, momentary emotional states among children. In contrast, disembodied CAs emerged as the predominant choice for ameliorating more stable emotional problems among adolescents and young adults. This nuanced understanding prompts a crucial consideration in the strategic deployment of CAs within the young population. Decisions regarding the selection of automated CA types should not only factor in age group distinctions but also align with the specific type of representation and emotional outcomes targeted by the intervention.

Feasibility and usability outcomes present an optimistic outlook, portraying automated CAs mediated interventions for youths’ emotional problems as generally acceptable and feasible, with high usability. Nevertheless, the implementation of automated CA interventions with youths encounters specific challenges. Firstly, automated CAs introduce potential safety risks, underscoring the imperative to address concerns related to suicidal ideation^12,26^. Second, engagement and adherence appear to decrease over time^29,31^. Third, the drop-out rate is overall higher than those reported in previous studies for other therapy formats^51^. These findings can be due to the fully automated nature of the CAs which acts as a self-help intervention. A review on the acceptability of online mental health programs for adolescents and young people found that drop-out rates were higher than the average when there was no concurrent therapist contact alongside digital components^52^. Although there is virtual guidance provided by the automated CA itself, it seems this might not be enough, and human assistance is needed besides the virtual assistance^52^. It is also possible that introducing youths to cutting-edge technology such as automated CAs may have a novelty effect, and that effect wears off in time, resulting in reduced engagement and adherence after prolonged interaction^14^.

Effectiveness remains inconclusive, challenging the assumption that technological advancements translate into improved efficacy. This finding is in accordance with some of the previous reviews conducted on evaluation of CAs in adult healthcare^14,53^. There are several potential explanations for these results. First, most of the automated CAs interventions reviewed here were in fact universal prevention, directed at youths from the general population, with initial low levels of mental health problems and consequently with limited room for improvement^54^. Indeed, when conducted on adults with clinical levels of anxiety or depression, a previous review showed medium to large effects of automated CAs interventions^13^. Second, according to a meta-analysis, for self-guided digital interventions to be efficient for youths, at least minimal support from a human therapist is needed^55^. The CAs mediated interventions included in the current paper were automated and, with only a few exceptions, standalone interventions, where the therapeutic agent was only the CA itself. Moreover, despite the limited evidence supporting their efficacy, a majority of automated CAs are commercially accessible, potentially emphasizing market accessibility over clinical efficacy. This incongruity underscores the imperative for a more robust evaluation of CAs’ effectiveness in addressing the mental health needs of the youth population.

The results of the current review must be interpreted in the light of several limitations. First, only studies published in peer reviewed journals were considered and it is possible that other automated CAs applications in gray literature, conference proceedings or other sources were not considered. Given the emerging status of the research in this area, it is plausible that a handful of ongoing studies are only published in conference proceedings. Second, the review focus was limited to the emotional component of mental health. Future reviews should consider the potential of automated CAs to address a wider range of clinical problems and symptoms, beyond those examined in our investigation. Small sample sizes, predominantly recruited from non-clinical populations are largely responsible for reduced generalizability of findings across many included articles. Therefore, a critical consideration for future research in the area is to enroll larger samples from the clinical population into trials to increase the power and generalizability of the findings. Fourth, there was a substantial heterogeneity in how the reported feasibility/usability and efficacy parameters were measured and conceptualized across studies, which makes findings hard to generalize. For example, engagement was defined in terms of subjective impressions on s attractiveness and enjoyment^27^, time spent per day or session in interacting with the automated CA^10^, percentage of target users returning for repeated sessions^37^, and number of exchanged messages with the application^43^. Similarly, efficacy outcomes such as anxiety and general distress were measured as salivary cortisol levels^8,41^, subjective feelings^27,35^, or in terms of behavioral cues^9^. Therefore, future research into automated CAs application would benefit from adhering to a standardized framework of measurement and conceptualization both in terms of feasibility/usability and evaluation outcomes to ensure comparability across studies.

Although most of the studies included measures of efficacy, usability or acceptability, there was no measurement of costs. Cost-effectiveness studies are needed to inform upon the affordability of such interventions in low and middle-income countries. Therefore, in our scoping review it was not possible to ascertain that automated CAs mediated psychological interventions are also cost effective when compared to the alternative approaches. Furthermore, more research on safety is warranted when speaking of fully automated CAs.

Another important direction would be to test whether integrating automated CAs as supporting the human therapist produces better results rather than just substituting it. Maybe a blended approach (face-to-face psychotherapy/ counseling) is the optimal solution for promoting mental health among youths while keeping the psychotherapeutic process engaging, attractive and safe at the same time. In addition, no comparison on feasibility, usability, or efficacy between different types of automated CAs was identified, despite preliminary results showing potential for differential responses to disembodied CAs, agents with virtual representation, and physical representation. It would be interesting to examine whether embodiment type predicts better engagement and clinical efficacy or is more preferred in a certain age group or context. Based on the existing research conducted on automated CAs we can’t generalize our findings to young people from low-income countries. Nonetheless, it is important to address this disparity through further investigations on the clinical efficacy of automated CAs with participants from different contexts, especially young people from low-income countries that face significant barriers in mental health treatment such as stigma, lack of financial resources, or lack of specialists.

Not lastly, we recommend involvement of end-users from early stages of development of automated CAs and changing the approach from developing automated CAs for youths to designing and devising them with youths, to enhance the uptake and acceptability of the application^50,51^. Additionally, the current state-of-the-art lacks information about the sustainability of effects; therefore, a more thorough investigation of usability and efficacy outcomes on long term is strongly recommended.

In conclusion, the field is characterized by a rapid expansion of use of fully automated CAs, with more and more evolved technical capabilities and especially in high income countries. Despite being highly acceptable, feasible and engaging as well as highly available for use, automated CAs do not appear to be yet prepared to be implemented in clinical practice with the young population. Although it is a promising approach for young population mental health promotion, efforts should be made to improve the efficacy and the safety of automated CAs. Future research with a standardized assessment, larger and diverse samples (e.g., different clinical conditions) and rigorous designs (e.g., efficacy and effectiveness studies, longer follow-ups) needs to be conducted.

## Methods

The scoping review was conducted in line with the PRISMA (Preferred Reporting Items for Systematic Reviews and Meta-Analyses) guidelines for conducting systematic scoping reviews^56^. The protocol for this scoping review was prospectively registered in OSF under the ID 10.17605/OSF.IO/8KU6P.

### Eligibility criteria

Inclusion criteria were: (1) primary studies based on either qualitative, quantitative, or mixed methods aiming to develop/design or test the usability, feasibility, efficacy, or economic cost effectiveness of a CA as a tool to improve a mental health outcome; (2) the CA is fully autonomous, meaning that it doesn’t rely on humans to generate responses or operate; (3) targeted samples of young population as end users, with a mean age ≤25 years; (4) published in a peer-reviewed journal and written in English. There were no inclusion restrictions on study design or on the mental or health status of participants.

Exclusion criteria were: (1) secondary research, conference proceedings, dissertations, and commentary articles aiming to describe or report on general aspects of human-CA interactions or interventions studies aimed to exclusively test general aspects of human-technology interactions using CAs; we also excluded studies describing or reporting on use of CA as a tool to improve social, cognitive, educational, or physical health outcomes as well as those focusing on CA applications for the purpose of assessment or monitoring only; (2) report on non-autonomous CAs, relying on a human user to generate responses (e.g., ‘Wizard of Oz’ methods) or semi-autonomous CAs, requiring a minimal human support to operate; (3) intervention targeted samples with a mean age >25; (4) written in languages other than English and published in gray literature.

### Search strategy

Systematic searches were conducted by RB in March 2023 in multidisciplinary as well as specific domain databases (Web of Science, PubMed, Scopus, PsychInfo, ACM -Association for Computing Machinery Digital Library and IEEE Xplore) and studies references using keywords related to conversational agents, the age of the population of interest, and the role/scope of intervention (see Supplementary Note 1 for a detailed sample of the search strategy).

### Study selection

The results of the search query were uploaded in EndNote (version 20; Clarivate Analytics). Following Cochrane recommendations, the screening process was piloted with a random sample of studies for both abstract and full text^57^. The articles were screened by the RB and CRP. Any disagreements between the 2 independent reviewers were resolved through consulting with AD.

### Data items and charting

A data form for exaction of information was designed prior to data charting and is detailed in the protocol for the current scoping review, published on Open Science Framework. The data extraction form was piloted and calibrated with the screening team. Like the study selection process, two reviewers (RB and CRP) independently conducted the process of data extraction, and any disagreements were resolved by the third reviewer (AD).

The following data items were charted: general information regarding the article (year, authors, country); technological characteristics (name, type of dialog system, availability, modality of communication, embodiment type); characteristics of the intervention (scope, mental health outcome targeted, duration, frequency, whether is standalone intervention and theoretical framework); characteristics of peer reviewed research (participants information, stage of research, study design and methodology and, if applicable, main results). A detailed overview of the definitions of each item together with corresponding categories is provided in Supplementary Table 3.

### Synthesis of the results

First, information about study meta-characteristics of articles as well as about landscape of the automated CAs’ based interventions, characteristics of research conducted in the area and technological characteristics of CA from data-charting were summarized using descriptive statistics and descriptive narration. Key findings from usability/feasibility and evaluation studies were tabulated and narratively summarized.

### Reporting summary

Further information on research design is available in the Nature Research Reporting Summary linked to this article.

### Supplementary information

Supplementary information

Reporting Summary

## Data Availability

The authors declare that the data supporting the findings of this study are available within the paper.

## References

[1] WHO. *World Health Statistics 2018:: Monitoring Health for the SDGs*. (WHO, 2018).

[2] Schlack R, Peerenboom N, Neuperdt L, Junker S, Beyer A-K (2021). The effects of mental health problems in childhood and adolescence in young adults: results of the KiGGS cohort. J. Health Monit..

[3] Aguirre Velasco A, Cruz ISS, Billings J, Jimenez M, Rowe S (2020). What are the barriers, facilitators and interventions targeting help-seeking behaviours for common mental health problems in adolescents? A systematic review. BMC Psychiatry.

[4] Wolters LH, Op de Beek V, Weidle B, Skokauskas N (2017). How can technology enhance cognitive behavioral therapy: the case of pediatric obsessive compulsive disorder. BMC Psychiatry.

[5] Laranjo L (2018). Conversational agents in healthcare: a systematic review. J. Am. Med. Inform. Assoc..

[6] Araujo T (2018). Living up to the chatbot hype: the influence of anthropomorphic design cues and communicative agency framing on conversational agent and company perceptions. Comput. Hum. Behav..

[7] Catania F, Spitale M, Garzotto F (2023). Conversational agents in therapeutic interventions for neurodevelopmental disorders: a survey. ACM Comput. Surv..

[8] Rossi S (2022). Using the Social Robot NAO for emotional support to children at a pediatric emergency department: randomized clinical trial. J. Med. Internet Res..

[9] Tanaka K, Hayakawa M, Noda C, Nakamura A, Akiyama C (2022). Effects of artificial intelligence aibo intervention on alleviating distress and fear in children. Child Adolesc. Psychiatry Ment. Health.

[10] Gabrielli S (2021). Engagement and effectiveness of a healthy-coping intervention via Chatbot for university students during the COVID-19 pandemic: mixed methods proof-of-concept study. JMIR MHealth UHealth.

[11] Kim Y, Thayne J, Wei Q (2017). An embodied agent helps anxious students in mathematics learning. *Educ*. Technol. Res. Dev..

[12] Nicol G, Wang R, Graham S, Dodd S, Garbutt J (2022). Chatbot-delivered cognitive behavioral therapy in adolescents with depression and anxiety during the COVID-19 pandemic: feasibility and acceptability study. JMIR Form. Res..

[13] Lim SM, Shiau CWC, Cheng LJ, Lau Y (2022). Chatbot-delivered psychotherapy for adults with depressive and anxiety symptoms: a systematic review and meta-regression. Behav. Ther..

[14] Kramer LL, Ter Stal S, Mulder BC, de Vet E, van Velsen L (2020). Developing embodied conversational agents for coaching people in a healthy lifestyle: scoping review. J. Med. Internet Res.

[15] Provoost S, Lau HM, Ruwaard J, Riper H (2017). Embodied conversational agents in clinical psychology: a scoping review. J. Med. Internet Res..

[16] Gaffney H, Mansell W, Tai S (2019). Conversational agents in the treatment of mental health problems: mixed-method systematic review. JMIR Ment. Health.

[17] Koulouri T, Macredie RD, Olakitan D (2022). Chatbots to support young adults’ mental health: an exploratory study of acceptability. ACM Trans. Interact. Intell. Syst..

[18] Bae Brandtzæg, P. B., Skjuve, M., Kristoffer Dysthe, K. K. & Følstad, A. When the social becomes non-human: young people’s perception of social support in chatbots. In *Proc. of the 2021 CHI Conference on Human Factors in Computing Systems* 1–13 (Association for Computing Machinery, New York, NY, USA,). 10.1145/3411764.3445318 (2021).

[19] Apolinário-Hagen J, Kemper J, Stürmer C (2017). Public acceptability of E-Mental health treatment services for psychological problems: a scoping review. JMIR Ment. Health.

[20] Car LT (2020). Conversational agents in health care: scoping review and conceptual analysis. J. Med. Internet Res..

[21] Kretzschmar K (2019). Can your phone be your therapist? Young People’s Ethical Perspectives on the Use of Fully Automated Conversational Agents (Chatbots) in Mental Health Support. Biomed. Inform. Insights.

[22] Ly KH, Ly A-M, Andersson G (2017). A fully automated conversational agent for promoting mental well-being: a pilot RCT using mixed methods. Internet Inter..

[23] Bartl-Pokorny KD (2021). Robot-based intervention for children with autism spectrum disorder: a systematic literature review. IEEE Access.

[24] Berrezueta-Guzman J, Robles-Bykbaev VE, Pau I, Pesántez-Avilés F, Martín-Ruiz M-L (2022). Robotic technologies in ADHD care: literature review. IEEE Access.

[25] Kabacińska K, Prescott TJ, Robillard JM (2021). Socially assistive robots as mental health interventions for children: a scoping review. Int. J. Soc. Robot..

[26] Bendig E, Erb B, Meißner D, Bauereiß N, Baumeister H (2021). Feasibility of a Software agent providing a brief Intervention for Self-help to Uplift psychological wellbeing (“SISU”). A single-group pretest-posttest trial investigating the potential of SISU to act as therapeutic agent. Internet Inter..

[27] Bray L (2020). The acceptability and impact of the Xploro digital therapeutic platform to inform and prepare children for planned procedures in a hospital: before and after evaluation study. J. Med. Internet Res..

[28] Dosovitsky G, Bunge E (2023). Development of a chatbot for depression: adolescent perceptions and recommendations. Child Adolesc. Ment. Health.

[29] Gabrielli S, Rizzi S, Carbone S, Donisi V (2020). A chatbot-based coaching intervention for adolescents to promote life skills: pilot study. JMIR Hum. Factors.

[30] Grové C (2020). Co-developing a Mental Health and Wellbeing Chatbot With and for Young People. Front. Psychiatry.

[31] He Y (2022). Mental health chatbot for young adults with depressive symptoms during the COVID-19 pandemic: single-blind, three-arm randomized controlled trial. J. Med. Internet Res..

[32] Høiland CG, Følstad A, Karahasanovic A (2020). Hi, can I help? Exploring how to design a mental health chatbot for youths. Hum. Technol..

[33] Jeong S (2023). Deploying a robotic positive psychology coach to improve college students’ psychological well-being. Use. Model. Use.Adapt. Interact..

[34] Kitt ER, Crossman MK, Matijczak A, Burns GB, Kazdin AE (2021). Evaluating the role of a socially assistive robot in children’s mental health care. J. Child Fam. Stud..

[35] Lappalainen P (2023). In the shadow of COVID-19: a randomized controlled online ACT trial promoting adolescent psychological flexibility and self-compassion. J. Context. Behav. Sci..

[36] Liu H (2022). chatbots to provide self-help depression interventions for university students: a randomized trial of effectiveness. Internet Inter..

[37] Ludin N (2022). A chatbot to support young people during the COVID-19 pandemic in New Zealand: evaluation of the real-world rollout of an open trial. J. Med. Internet Res..

[38] Russell JK, Strodl E, Kavanagh D (2021). Use of a social robot in the implementation of a narrative intervention for young people with cystic fibrosis: a feasibility study. Int. J. Soc. Robot..

[39] Trappey AJC, Lin APC, Hsu KYK, Trappey CV, Tu KLK (2022). Development of an empathy-centric counseling chatbot system capable of sentimental dialogue analysis. Processes.

[40] Williams R (2021). 21-day stress detox: open trial of a universal well-being chatbot for young adults. Soc. Sci..

[41] Crossman MK, Kazdin AE, Kitt ER (2018). The influence of a socially assistive robot on mood, anxiety, and arousal in children. Prof. Psychol. Res. Pract..

[42] Fitzpatrick KK, Darcy A, Vierhile M (2017). Delivering cognitive behavior therapy to young adults with symptoms of depression and anxiety using a fully automated conversational agent (Woebot): a randomized controlled trial. JMIR Ment. Health.

[43] Fulmer R, Joerin A, Gentile B, Lakerink L, Rauws M (2018). Using psychological artificial intelligence (Tess) to relieve symptoms of depression and anxiety: randomized controlled trial. JMIR Ment. Health.

[44] Greer S (2019). Use of the Chatbot ‘Vivibot’ to deliver positive psychology skills and promote well-being among young people after cancer treatment: randomized controlled feasibility trial. JMIR MHealth UHealth.

[45] Okita SY (2013). Self-other’s perspective taking: the use of therapeutic robot companions as social agents for reducing pain and anxiety in pediatric patients. Cyberpsycho. Behav. Soc. Netw..

[46] Global Smartphone Market Analysis and Outlook: Disruption in a Changing Market - PDF Free Download. https://docplayer.net/1973753-Global-smartphone-market-analysis-and-outlook-disruption-in-a-changing-market.html.

[47] Schuetzler RM, Giboney JS, Grimes GM, Nunamaker JF (2018). The influence of conversational agent embodiment and conversational relevance on socially desirable responding. Decis. Support Syst..

[48] Nguyen, H. Examining teenagers’ perceptions of conversational agents in learning settings. In *Interaction Design and Children* 374–381 (ACM, Braga Portugal,). 10.1145/3501712.3529740 (2022).

[49] Michel T, Tachtler F, Slovak P, Fitzpatrick G (2020). Young People’s attitude toward positive psychology interventions: thematic analysis. JMIR Hum. Factors.

[50] Harte R (2017). A human-centered design methodology to enhance the usability, human factors, and user experience of connected health systems: a three-phase methodology. JMIR Hum. Factors.

[51] Wight D, Wimbush E, Jepson R, Doi L (2016). Six steps in quality intervention development (6SQuID). J. Epidemiol. Community Health.

[52] Struthers A (2015). The acceptability of E-mental health services for children, adolescents, and young adults: a systematic search and review. Can. J. Commun. Ment. Health.

[53] Bendig E, Erb B, Schulze-Thuesing L, Baumeister H (2019). The next generation: chatbots in clinical psychology and psychotherapy to foster mental health – a scoping review. Verhaltenstherapie.

[54] Werner-Seidler A, Perry Y, Calear AL, Newby JM, Christensen H (2017). School-based depression and anxiety prevention programs for young people: a systematic review and meta-analysis. Clin. Psychol. Rev..

[55] Bennett SD (2019). Practitioner review: unguided and guided self-help interventions for common mental health disorders in children and adolescents: a systematic review and meta-analysis. J. Child Psychol. Psychiatry.

[56] Tricco AC (2018). PRISMA Extension for Scoping Reviews (PRISMA-ScR): checklist and explanation. Ann. Intern. Med..

[57] Garritty C (2021). Cochrane rapid reviews methods group offers evidence-informed guidance to conduct rapid reviews. J. Clin. Epidemiol..

