# Supplementary material for: Use of automated conversational agents in improving young population mental health: a scoping review
Source: NPJ Digit Med. 2024 Mar 19;7:75. doi: 10.1038/s41746-024-01072-1 (PMC10951258; doi:10.1038/s41746-024-01072-1)
Supplement: Supplementary file 1 — Supplementary information [file 41746_2024_1072_MOESM1_ESM.pdf]

Supplementary Table 1. Characteristics of CAs and interventions

			**Characteristics of CAs**						**Characteristic of interventions**		
Study and year publication	Country	CA name	Dialog system	Communication modality	Type of embodiment	Availability	MH targeted	Scope	Duration/ frequency	Standalone vs. component	Theoretical framework
Bending 2021	Germany	SISU	Rule-based	Text	Disembodied	No	Psychological well being	Prevention	2 days/daily	Standalone	ACT
Bray 2020	US	Xploro	AI-based	NR	Virtual	Yes	Medical procedures related anxiety	Intervention	NR	Component	NR
Crossman 2018	US	Paro	AI-based	Non-verbal	Physical	Yes	Mood Anxiety	Intervention	15 minutes	Standalone	NR
Dosovitsky 2023	US	BethBot	Rule-based	Text	Disembodied	No	Depression	Prevention	1 session	Standalone	CBT
Fitzpatrick 2017	US	Woebot	Mixed	Text	Disembodied	Yes	Depression Anxiety	Intervention	2 weeks (up to 20 sessions)	Standalone	CBT
Fulmer 2018	US	Tess	AI-based	Text	Disembodied	Yes	Depression Anxiety	Prevention	2 weeks/daily; 4 weeks/bi-weekly	Standalone	CBT; TM; EFT; MI
Gabrielli 2021	Italy	Atena	Rule-based	Text	Disembodied	Yes	Distress Anxiety	Prevention	4 weeks/bi-weekly	Standalone	CBT; PP
Gabrielli 2020	Italy	CRI/CRIS	Rule-based	Text	Disembodied	No	Psychological well being	Prevention	4 weeks/bi-weekly	Standalone	NR
Greer 2019	US	Vivibot	Rule-based	Text	Disembodied	Yes	Psychological well being	Prevention	4 weeks	Standalone	CBT; PP
Grové 2021	Australia	Aish	AI-based	Text	Disembodied	No	Psychological well being	Prevention	NR	Standalone	NR
He 2022	China	XiaoE	AI-based	Multimodal	Disembodied	Yes	Depression	Intervention	1 week	Standalone	CBT
Høiland 2020	Norway	Health Buddy	Rule-based	Text	Disembodied	No	Mental health problems	Prevention	NR	Standalone	NR

		Characteristics of CAs						Characteristic of interventions			
Study and year publication	Country	CA name	Dialog system	Communication modality	Type of embodiment	Availability	MH targeted	Scope	Duration/ frequency	Standalone vs. component	Theoretical framework
Jeong 2022	US	Jibo	Rule-based	Multimodal	Physical	Yes	Psychological well being<br>Mood	Prevention	1 week/ daily	Standalone	PP
Kim 2017	US	Chris	Rule-based	Multimodal	Virtual	No	Mathematics anxiety	Prevention	NR	Component	CBT
Kitt 2022	US	Paro	AI-based	Non-verbal	Physical	Yes	Distress	Prevention	3 minutes	Standalone	NR
Lappalainen 2023	Finland	NR	Rule-based	Text	Disembodied	No	Anxiety<br>Depression	Prevention	5 weeks/3 times a week	Component	ACT
Liu 2022	China	XiaoNan	Mixed	Multimodal	Disembodied	Yes	Depression	Intervention	16 weeks	Standalone	CBT
Ludin 2022	New Zeeland	Aroha	Mixed	Test	Disembodied	Yes	COVID related anxiety	Prevention	NR	Standalone	CBT; PP
Nicol 2022	US	Woebot-Wgen Z	AI-based	Text	Disembodied	Yes	Depression<br>Anxiety	Intervention	12 weeks	Component	CBT, IPT; DBT
Okita 2013	US	Paro	AI-based	Non-verbal	Physical	Yes	Anxiety	Prevention	30 minutes	Standalone	NR
Rossi 2022	Italy	NAO	AI-based	Speech	Physical	Yes	Anxiety	Prevention	NR	Standalone	CBT
Russel 2021	US	NAO	AI-based	Speech	Physical	Yes	Anxiety<br>Depression	Prevention	2 weeks/ 3 sessions	Standalone	MINI
Tanaka 2022	Japan	Aibo	AI-based	Non-verbal	Physical	Yes	Distress	Prevention	5 minutes	Standalone	NR
Trappey 2022	Taiwan	NR	AI-based	Multimodal	Disembodied + Virtual	-	Distress	Intervention	2 weeks/once a week	Component	PCT
William 2021	New Zeeland	21 Days Stress Detox	Rule-based	Text	Disembodied	Yes	Distress	Intervention	3 weeks	Standalone	CBT; PP

Notes: NR – not reported; ACT – Acceptance and Commitment Therapy; CBT – Cognitive Behavioral Therapy; PP- Positive Psychology; TM – Transtheoretical Model; EFT – Emotion focused therapy; MI – Motivational Interview; DBT – Dialectical and Behavioral Therapy; IPT – Interpersonal Therapy; MINI – Metacognitive Intervention of Narrative Imagery PCT – Person Centered Therapy.

Supplementary Table 2. Characteristics of peer reviewed research

Study	Recruitment setting	Health status	Sample size	% female	Age range/ mean age	Drop out (%)	Stage research	Study design and methodology	Type of control	Usability/Feasibility outcomes	Evaluation outcomes
Bending 2021	Community	Any	30	80%	M=23.17	0	Feasibility/ usability and evaluation	Uncontrolled pre post evaluation<br><br>Mixed methods	NA	Functionality: High functionality; Acceptability: High acceptability - Attitudes and expectations were comparable to those towards psychological internet interventions in general; Positive user experience	Anxiety ↑<br>Depression ↔<br>PTSD ↔
Bray 2020	Hospital	Undergoing a medical procedure	80	EC 60% CG 50%	8-14<br>M_{CG} =10.4;<br>M_{EG} =12	0	Feasibility/ usability and evaluation	Non-randomized controlled trial<br><br>Mixed methods	AC- standard care	Engagement: intervention rated as enjoyable, funny, and easy to use	Procedural medical anxiety ↓ only for those more frequently exposed to medical procedures and undergoing invasive medical procedure
Crossman 2018	Community	Any at recruitment – experimental induced stress	87	52.9%	6-9<br>M=8.15	17	Evaluation	Randomized controlled trial<br><br>Quantitative	AC – non robotic: PC (WL)	NA	Anxiety ↔<br>Physiological arousal ↔<br>Negative affect ↔<br>Positive affect ↑
Dosovitsky 2023	Community	Any	23	40.9%	13-18<br>(M=14.96)	44	Feasibility/ usability	Uncontrolled post evaluation<br><br>Mixed methods	NA	Retention: Over half (56.5%) of the sample completed the full intervention: User experience: 54% of users rated the experience of using the chatbot as positive	NA
Fitzpatrick 2017	Educational	Self-reported anxiety or depression symptoms	70	67%	18-28<br>(M=22.2)	17	Feasibility/ usability and evaluation	Randomized controlled trial<br><br>Mixed methods	AC - information	Engagement: Participants in EG checked in with the bot an average of 12.14 times, over the 2-week period; User satisfaction: Significantly higher levels of satisfaction both overall and with content, significantly greater amount of emotional awareness because of using the bot than the CG. Learnability: All (100%) of the participants in EG endorsed having learned something new versus three-quarters (77%) of the CG.	Depression ↓<br>Anxiety ↔<br>Positive affect ↔<br>Negative affect ↔
Fulmer 2018	Educational	Any	74	70%	M=22.9	0 CG;<br>1 EG	Feasibility/ usability and evaluation	Randomized controlled trial<br><br>Mixed methods	AC – information	User satisfaction: Higher satisfaction in EG compared to CG Engagement: higher engagement in EG compared to CG	Depression ↓<br>Anxiety ↓<br>Positive affect ↑<br>Negative affect ↓

Gabrielli 2021	Educational	Any	71	67%	M=20.6	42	Feasibility/ usability and evaluation	Uncontrolled pre post evaluation<br><br>Mixed methods	NA	Attrition: Overall attrition was 42%; drop out increased after 2 weeks of utilization of the chatbot. Engagement: Participants interacted with the CA an average of 78 times (SD 24.8; median 81; range 5-158) over the 4-week period. The average number of uncompleted sessions was 3.1 (SD 2.3) out of 8 overall sessions. Usability: High overall perceived usability, but neutral attitudes toward the aspect of CA and rewarding experience	Anxiety ↓only for those with high levels of anxiety<br>Distress ↓ for those with initial high stress scores<br>Distress ↑ for those with initial low stress levels
Gabrielli 2020	NR	Any	21	38%	12-17 (M=14.52)	0	Developmen t and feasibility/ usability	Phase 1. Co-participatory design<br>Phase 2 – Uncontrolled post evaluation<br><br>Mixed methods	NA	User satisfaction: Most of the participants found the intervention useful (16/21, 76%), easy to use (19/21, 90%), and innovative (17/21, 81%). They also thought that a session should last only 5-10 minutes (14/21, 66%) and said they would recommend the intervention to a friend (20/21, 95%).	NA
Greer 2019	Community	Cancer diagnosis and completing treatment for cancer within last 5 years	45	80%	25.00	37	Feasibility/ usability and evaluation	Randomized controlled trial - pilot<br><br>Mixed method	AC – daily emotional ratings	Retention: Survey completion was 73% at 2 and 4 weeks, and 58% (26/45) at 8 weeks Engagement: EG spent an average of 73.8 (SD 52) min across an average of 12.1 (SD 7.1) engaged sessions chatting with CA vs. CG 27.13 (SD 15.8) min across an average of 18.1 (SD 8.6). Perceived helpfulness: Participants rated their experience with CA as helpful (mean 2.0/3, SD 0.72) and would recommend it to a friend.	Anxiety ↓<br>Depression ↔<br>Positive affect ↔<br>Negative affect ↔
Grové 2021	NA	NA	NA	NA	NA	NA	Design and Developmen t	Co-participatory design	NA	NA	NA

He 2022	Educational	Self-reported depression symptoms	148	37.2%	M=18.78	47	Feasibility/ usability and evaluation	Randomized controlled trial<br><br>Mixed methods	AC – CG1 – Ebook CG2 – chatbot not mental health related	Attrition: significantly higher in CGs compared to EG (37% vs. 10%). Engagement: participants in the EG interacted with the chatbot for 25.54 sessions (range 0-172) on average per day, and each session lasted an average of 22.46 seconds (range 0-758 seconds) over the 1-week period. The daily frequency and duration of the interaction were high on day 1, day 2, and day 7, while they were relatively low on day 3, day 5, and day 6, and rebounded to some extent on day 4. The frequency of the interaction reached peaks in the 3 time periods of 8-10 AM, 12-2 PM, and 4-6 PM per day. Acceptability: Better acceptability (AS; $F_{2,145}=4.322$; $P=.02$) were discovered with EG Usability: no significant difference between EG and CGs	Depression ↓ at T1<br>Depression ↓ at T2 compared to CG2, but ↔ at T2 compared to CG1
Høiland 2020	NA	NA	NA	NA	NA	NA	Design and development	Co-participatory design<br><br>Qualitative	NA	NA	NA
Jeong 2022	Educational	Any	42	64.29 %	18.94	NR	Feasibility/usability and evaluation	Uncontrolled pre post evaluation<br><br>Mixed methods	NA	Acceptability: positive ratings of content of intervention; negative ratings for duration – preference for longer sessions; overall positive interaction with robot; negative rating of system confidentiality and technic functioning	Psychological well being ↑<br>Overall mood ↑
Kim 2017	Educational	Any	138	53.6%	M=15.91	NR	Evaluation	Randomized controlled trial<br><br>Quantitative	PC – instructional messages without anxiety messages	NA	Mathematics anxiety ↔
Kitt 2021	Community	Any	70	57.1%	M=8.76	NR	Evaluation	Randomized controlled trial<br><br>Quantitative	PC – no intervention	NA	Negative affect ↔<br>Positive affect ↑
Lappalainen 2023	Educational	Any	234	66.7%	14-16 (M=15.01)	41.5	Evaluation	Randomized controlled trial<br><br>Quantitative	AC – student coach + virtual coach<br>PC – no intervention	NA	Anxiety ↔<br>Depression ↔

Liu 2022	Educational	Self-reported depression	83	55.42 %	19-28 (M=23.08)	24.10	Feasibility/ usability and evaluation	Randomized controlled trial<br><br>Mixed methods	AC bibliotherap y	Adherence: decreased adherence in EG vs. CG; User satisfaction: no significant difference between EG and CG	Anxiety ↓<br>Depression ↓<br>Positive affect ↔<br>Negative affect ↔
Ludin 2022	Community	Any	127	70.9%	13-14	70.9	Developmen t feasibility/ usability and evaluation	Open trial<br><br>Mixed methods	NA	Initial uptake: In the 2 weeks following the launch of the chatbot and the open trial, there were 393 registrations, and 238 users logged into the chatbot. Engagement: Target users engaged with CA for 11 minutes, 31 returned for repeat sessions. Adherence/retention: 30 users (out of 81, 37%) who completed the pre-post measure of COVID-19 anxiety, both in the initial session and the outro.	Covid related anxiety ↓
Nicol 2022	Hospital (Primary pediatric care)	Diagnosis of depression and anxiety in the past 3 months	18	88%	13-17 (M=14.7)	5.5	Feasibility/ usability and evaluation	Randomized controlled trial - pilot<br><br>Mixed methods	PC – WL	Acceptability. feasibility (possible to use) and system usability: High acceptability, feasibility, and usability of CA Safety: 10 (59%) of participants, of which 4 (40%) were in the EG and 6 (35%) were in CG, triggered at least 1 alarm to assess for suicidal ideation. During study participation, 4 (24%) of the participants had 1 alert, 4 (24%) had 3, and 2 (12%) had 6. One parent from the EG reported at week 12 that their teen was seen in an ED and discharged to home	Anxiety ↓<br>Depression ↓
Okita 2013	Hospital	Any pathological health condition	18	100%	6-16	NR	Evaluation	Randomized controlled trial Quantitative	AC - C only vs C and P	NA	Negative emotional anxiety trait ↓; Positive emotional anxiety trait ↔
Rossi 2022	Hospital	Any health condition presenting to pediatric emergency room	109	48.9%	5-8	13.76	Evaluation	Randomized controlled trial<br><br>Quantitative	AC – play with nurse<br>PC – No intervention	NA	Distress<br>↓ 20 min after intervention<br>↔ immediately after intervention

Russel 2021	Hospital	cystic fibrosis	8	37.5%	10-14 (M=11.5)	NR	Feasibility /usability and evaluation	Uncontrolled pre post evaluation<br><br>Quantitative	NA	Acceptability: positive view of the interactions with the CA; acceptability rate of 100%, i.e., all eight participants had an overall positive impression (≤ 5) of the robot.	Anxiety ↓<br>Depression minimal ↓
Tanaka 2022	Hospital	Undergoing vaccine procedures	53	EG= 69%<br>CG=44 %	3-12 (M_{EG}= 4.41 M_{CG}=3.96)	0	Evaluation	Non-randomized controlled trial<br><br>Quantitative	AC – non robotic control	NA	Distress<br>Immediately after intervention ↔<br>5 min post intervention ↓
Trappey 2022	Educational	High level of self-reported stress	34	50%	20-26 (M=22.76)	NR	Design and evaluation	Uncontrolled pre post evaluation<br><br>Mixed methods	NA	NA	Distress ↓<br>Psychological sensitivity ↓
Williams 2021	Educational	Self-identified as stressed	124	81%	18-24	51.6	Feasibility/ usability and evaluation	Uncontrolled pre post evaluation<br><br>Mixed methods	NA	Adherence: On average, participants adhered to the program for 11 days out of the total 21-day program (M = 11.3, SD = 7.8). 30 participants (27.3%) adhered fully, completing all 21 days (and/or up to day 22 which was content-free); 15 (13.6%) completed between 15–20 days; 11 (10%) completed between 10–14 days; 23 (20.9%) did 5–9 days; and 25 22.7%) did between two and four days of content. Six (5.5%) discontinued after day one. Acceptability: Over 90% of participants who gave a chatbot rating during the program rated their experience as "Okay" or "Great".	Wellbeing ↑<br>Distress ↓<br>Anxiety ↔<br>Subjective happiness ↔

Supplementary Table 3. Categories, components and definitions used for data extraction and categorization

Category	Definition and components
Bibliographical information	
First Author	The first author of the article
Year of publication	The year of publication
Country	Country where study was conducted or country of authors (for design and development studies)
Technological features	
Name CA	The name which is given to the specific CA
Dialog system	The type of dialog system underlying the process of conversation<br>• Rule-based CA- conversational agents whose dialogue or interactions were predefined but assembled and matched to the user input in a dynamic manner.<br>• Artificial intelligence- based CA - conversational agents that employ natural language processing and machine learning to carry on a interaction<br>• Mixed CA - combines predefined assembled dialogue with natural language processing and machine learning to carry on a interaction
Modality of communication	The modality of communication as input and output<br>Text – uses written text as mean of communication<br>Voice – uses voice speech as mean of communication<br>Non-verbal – use non-verbal cues as means of communication (e.g., gestures, facial expression, touching)<br>Multimodal - combination of above-mentioned modalities
Availability	Whether the conversational agent described in the study may be commercially or acquired for personal use, independently of the study
Embodiment type	The visual representation of the agent<br>• Disembodied CA (chatbot)<br>• CA with virtual representation<br>• CA with physical representation (robot)
Characteristics of interventions	

Mental health condition/aspect	The mental health outcome targeted by the intervention
Scope of intervention	The aim of the intervention based on the mental health - illness conceptualization<br><br>● Prevention programs aimed at reducing the likelihood of future mental health conditions/difficulties in the general population or for people who are identified as being at risk of a mental health condition.<br><br>● Targeted interventions - delivered to young people who have detectable mental health problems (based on screening) or have a diagnosed mental disorder.
Duration and frequency of intervention	Duration of intervention in days/weeks and frequency of intervention
Independency	Whether the conversational agent is an independent intervention (standalone) or is a integrated component of another intervention<br>● Standalone intervention<br>● Integrated component
Theoretical framework	The conceptual approach that informed the development of psychological intervention, as stated by authors (e.g., Cognitive Behavioral Theory, Problem Solving, Transactional Model of Coping)
Characteristics of peer reviewed research	
Stage of research	The stage of research progress related to the complex development of intervention<br><br>● *Design and development studies* refer to articles describing systems design, or the development of conversational agents, without reporting data on their effectiveness, their usability, acceptability, or feasibility.<br><br>● *Feasibility/Usability* includes articles describing the results of pilot/feasibility studies focusing on process and procedural outcomes (eg, acceptability, participation, utilization, retention and recruitment, adherence, or compliance), or on technical parameters of the system design (e.g., user experience, satisfaction with the digital system, ease-of-use, task-efficiency, usefulness, learnability, memorability, engagement) rather than on the effects on the targeted problem.<br>(Gould, 1985).<br><br>● *Evaluation* includes studies reporting on the effects of the CA based interventions on targeted mental health aspects and related psychological outcomes relevant to the scope of the intervention.<br><br>Evaluations studies include:<br>1. Effectiveness/efficacy studies

	● Controlled studies (Randomized controlled studies, non-randomized controlled study)<br>● Uncontrolled studies (pre-post evaluation, post evaluation only)<br>2. Economic evaluation – assess the costs or cost-effectiveness of the CA intervention<br><br>Since it is expected that some articles will cover a combination of the stages of research, combined categories will also be used (e.g., design, usability/feasibility, and efficacy; usability/ feasibility and evaluation etc.).
Study design and methodology	Type of methodology (quantitative, qualitative, mixed), design of the study (controlled – randomized, nonrandomized; uncontrolled pre-post, post only) and type of control for controlled studies (active vs. passive control)
Outcomes and main results	The types of outcomes reported as described in stage of research<br>● Feasibility/Usability outcomes (e.g., participation, acceptability, adherence, retention, user satisfaction, engagement, learnability, ease of use, positive and negative aspects of using the conversational agent)<br>● Evaluation outcomes (e.g., depression, anxiety, procedural anxiety, speaking anxiety, stress, positive and negative mood)<br><br>Main findings related to the outcomes
Recruitment setting	Setting from where participants in the study are recruited (e.g., educational, hospital, community etc)
Health status	The mental and physical health status of participants with whom the CA is intended to be used (e.g., any, youths with a health condition (cancer), youths with a mental health condition (depressed patients)
Sample size	The number of participants in the study
Mean age	Mean age of sample
%female	Gender distribution expressed as percentage of females
Drop-out rate	The estimate of number of subjects that left out the study before completion due to some reason

Supplementary Note 1. Search string sample

Web of Science (WOS)

(TS=("Artificial intelligence chatbot" OR "AI agent" OR chatterbox OR chatbot OR chatterbot OR "Conversational agent" OR "Conversational system" OR "Conversational assistant" OR "Conversational User Interface" OR "Conversational interface" OR "Conversational AI" OR "Dialog system" OR "virtual agent" OR "virtual assistant" OR "Intelligent agent" OR smartbot OR "Talking avatar" OR "Virtual coach" OR robot OR "embodied agent" OR "avatar agent" OR "virtual avatar" OR "embodied avatar") AND TS=("teenagers" OR "teens" OR "youth" OR "juvenile*" OR "young adults" OR "young people" OR "young person" OR "students" OR "University" OR "Universities" OR "adolescents" OR "adolescence" OR "young people" OR "child" OR "children" OR "childhood")) AND TS=(intervention OR prevention OR prevent OR reduce OR reduction OR promote OR promoting OR treat OR treatment OR improve OR program OR counsel OR counseling)
