# Supplementary material for: Use of automated conversational agents in improving young population mental health: a scoping review
Source: NPJ Digit Med. 2024 Mar 19;7:75. doi: 10.1038/s41746-024-01072-1 (PMC10951258; doi:10.1038/s41746-024-01072-1)
Supplement: Supplementary file 2 — Reporting Summary [file 41746_2024_1072_MOESM2_ESM.pdf]

nature portfolio

Corresponding author(s): BLPR NPJDIGITALMED-07815
Last updated by author(s): 19.08.2023

Reporting Summary

Nature Portfolio wishes to improve the reproducibility of the work that we publish. This form provides structure for consistency and transparency in reporting. For further information on Nature Portfolio policies, see our Editorial Policies and the Editorial Policy Checklist.

Please do not complete any field with "not applicable" or n/a. Refer to the help text for what text to use if an item is not relevant to your study.
For final submission: please carefully check your responses for accuracy; you will not be able to make changes later.

Statistics

For all statistical analyses, confirm that the following items are present in the figure legend, table legend, main text, or Methods section.

n/a	Confirmed	
X	☐	The exact sample size (n) for each experimental group/condition, given as a discrete number and unit of measurement
X	☐	A statement on whether measurements were taken from distinct samples or whether the same sample was measured repeatedly
X	☐	The statistical test(s) used AND whether they are one- or two-sided<br>*Only common tests should be described solely by name; describe more complex techniques in the Methods section.*
X	☐	A description of all covariates tested
X	☐	A description of any assumptions or corrections, such as tests of normality and adjustment for multiple comparisons
X	☐	A full description of the statistical parameters including central tendency (e.g. means) or other basic estimates (e.g. regression coefficient) AND variation (e.g. standard deviation) or associated estimates of uncertainty (e.g. confidence intervals)
X	☐	For null hypothesis testing, the test statistic (e.g. F, t, r) with confidence intervals, effect sizes, degrees of freedom and P value noted<br>*Give P values as exact values whenever suitable.*
X	☐	For Bayesian analysis, information on the choice of priors and Markov chain Monte Carlo settings
X	☐	For hierarchical and complex designs, identification of the appropriate level for tests and full reporting of outcomes
X	☐	Estimates of effect sizes (e.g. Cohen's d, Pearson's r), indicating how they were calculated

Our web collection on statistics for biologists contains articles on many of the points above.

Software and code

Policy information about availability of computer code

Data collection	Not applicable
Data analysis	Not applicable

For manuscripts utilizing custom algorithms or software that are central to the research but not yet described in published literature, software must be made available to editors and reviewers. We strongly encourage code deposition in a community repository (e.g. GitHub). See the Nature Portfolio guidelines for submitting code & software for further information.

Data

Policy information about availability of data

All manuscripts must include a data availability statement. This statement should provide the following information, where applicable:
- Accession codes, unique identifiers, or web links for publicly available datasets
- A description of any restrictions on data availability
- For clinical datasets or third party data, please ensure that the statement adheres to our policy

This article is a scoping review from the publicly available studies and all extracted data are included in the review.

Research involving human participants, their data, or biological material

Policy information about studies with human participants or human data. See also policy information about sex, gender (identity/presentation), and sexual orientation and race, ethnicity and racism.

Reporting on sex and gender

We used gender inclusive terms through the manuscript. Even if we aimed at presenting the gender in a more nuanced way, the primary studies did not include detailed information on this aspect.

Reporting on race, ethnicity, or other socially relevant groupings

We did not make any reporting based on race, ethnicity, or other socially relevant groupings since it was not within the aim of the review.

Population characteristics

We only performed narrative synthesis of use of conversational agents in improving young population mental health. We did not extract and use any individual participants characteristics. However, characteristics of participants were clearly described in the study. The 25 studies included in the final review included 1707 children, adolescents, and young adults with a maximum mean age of 25 years. Participants were recruited predominantly from community settings, with only 7 studies including participants with elevated levels of emotional problems.

Recruitment

Not applicable

Ethics oversight

Not applicable

Note that full information on the approval of the study protocol must also be provided in the manuscript.

Field-specific reporting

Please select the one below that is the best fit for your research. If you are not sure, read the appropriate sections before making your selection.

☐ Life sciences ☒ Behavioural & social sciences ☐ Ecological, evolutionary & environmental sciences

For a reference copy of the document with all sections, see nature.com/documents/nr-reporting-summary-flat.pdf

Behavioural & social sciences study design

All studies must disclose on these points even when the disclosure is negative.

Study description

The current study aimed to conduct a scoping review on automated conversational agents use in improving mental health among children, adolescents, and young adults. We were interested in describing the characteristics of conversational agents, Interventions and current evidence on development, usability/feasibility, and effectiveness/cost-effectiveness.

Research sample

Sample size of the 25 primary studies included in the review consisted of 1707 participants, children, adolescents, And young adults with a mean age of max. 25 years old. Females represented 58% of the total sample. Most of the studies reported on participants recruited from community population.

Sampling strategy

Not applicable

Data collection

We conducted systematic searches in the databases using key words related to domain of interest.

The data presented in the review were extracted from the primary articles included.

Timing

Not applicable

Data exclusions	Secondary research, conference proceedings, dissertations, protocols, and commentary articles aiming to describe or report on general aspects of human-CA interactions, use of non-automated CA as a tool for assessment, monitoring or to improve other outcomes than the mental health component of emotional regulation and coping with events were excluded. Studies that focused on use of CA mediated interventions in adults over 25 years old (Mean age>25) were also excluded.
Non-participation	Not applicable
Randomization	Not applicable

Reporting for specific materials, systems and methods

We require information from authors about some types of materials, experimental systems and methods used in many studies. Here, indicate whether each material, system or method listed is relevant to your study. If you are not sure if a list item applies to your research, read the appropriate section before selecting a response.

Materials & experimental systems

n/a	Involved in the study
X	☐ Antibodies
X	☐ Eukaryotic cell lines
X	☐ Palaeontology and archaeology
X	☐ Animals and other organisms
X	☐ Clinical data
X	☐ Dual use research of concern
X	☐ Plants

Methods

n/a	Involved in the study
X	☐ ChIP-seq
X	☐ Flow cytometry
X	☐ MRI-based neuroimaging

This checklist template is licensed under a Creative Commons Attribution 4.0 International License, which permits use, sharing, adaptation, distribution and reproduction in any medium or format, as long as you give appropriate credit to the original author(s) and the source, provide a link to the Creative Commons license, and indicate if changes were made. The images or other third party material in this article are included in the article's Creative Commons license, unless indicated otherwise in a credit line to the material. If material is not included in the article's Creative Commons license and your intended use is not permitted by statutory regulation or exceeds the permitted use, you will need to obtain permission directly from the copyright holder. To view a copy of this license, visit http://creativecommons.org/licenses/by/4.0/
